# Interventions for stress, burnout, and resilience in dental and oral health students: a systematic review

**DOI:** 10.3389/fmed.2026.1945728

**Published:** 2026-09-07

**Authors:** Xiaohong Deng, Wenjuan Qiang, Tiezhou Hou, Le Qiang

**Affiliations:** 1 Key Laboratory of Shaanxi Province for Craniofacial Precision Medicine Research, College of Stomatology, Xi’an Jiaotong University, Xi'an, China; 2 Clinical Research Center of Shaanxi Province for Dental and Maxillofacial Diseases, College of Stomatology, Xi’an Jiaotong University, Xi'an, China; 3 Department of Cariology and Endodontics, College of Stomatology, Xi’an Jiaotong University, Xi'an, China

**Keywords:** burnout, dental students, mindulness, psychological stress, resillience

## Abstract

**Background:**

Dental and oral health students experience substantial stress, yet the intervention evidence published over the past decade has not been comprehensively synthesized.

**Methods:**

We searched MEDLINE/PubMed, Embase, Scopus, Web of Science Core Collection, CENTRAL, ERIC, and PsycINFO for reports published from January 2015 through June 2026. Randomized, non-randomized controlled, crossover, and uncontrolled pre–post studies were eligible if they evaluated an intervention and reported a validated perceived-stress, psychological-distress, burnout, or resilience outcome. Risk of bias was assessed with RoB 2, ROBINS-I, and design-specific criteria for uncontrolled studies. Synthesis followed SWiM and prioritized direction and magnitude, design, sample size, and precision rather than statistical significance alone; certainty was rated with GRADE.

**Results:**

Fourteen studies from 11 countries reported study sample sizes totaling 750 participants (median 47.5; range 5–120), including 102 participants from one mixed health-professions trial in which dentistry-specific outcomes were not separable; outcome-specific denominators were often smaller. Three were explicitly randomized controlled trials, one quasi-experimental study reported random group allocation, four used other controlled or crossover designs, and six were uncontrolled. Interventions comprised mindfulness or meditation (four studies), coaching/counseling/cognitive-behavioral or psychoeducational approaches (five), movement or sensory/relaxation approaches (three), and resilience training (two). Within-group improvements were common, but comparative findings were mixed: several apparent benefits arose from very small, self-selected, or high-attrition samples. One uncontrolled study (*n* = 69) directly measured burnout and found immediate reductions in personal and study-related domains, whereas colleague- and teacher-related domains did not change. Long-term follow was uncommon. Certainty was very low for perceived stress/psychological distress, burnout, and resilience.

**Conclusions:**

Student-facing programs may improve selected short-term perceived-stress, psychological-distress, coping, or burnout outcomes, but durable effectiveness is unestablished. Confidential counseling/referral and brief, supported skills modules appear feasible as evaluated adjuncts; stand-alone digital or sensory interventions and claims of burnout prevention should remain experimental. These programs should not substitute for organization-level prevention.

**Systematic Review Registration:**

https://www.crd.york.ac.uk/PROSPERO/view/CRD420261449695.

## 1 Introduction

Dental education combines dense biomedical curricula, repeated high-stakes assessment, fine-motor performance, and early responsibility for patient care. These demands help explain why perceived stress, psychological distress, and burnout are repeatedly reported across dental schools (1–7). Burnout is not synonymous with transient stress: it reflects a sustained pattern of exhaustion, distancing or cynicism, and reduced efficacy arising in an occupational or study context (8, 9). Conflating these constructs can make a short-term change in perceived stress look like evidence that burnout has been prevented when it has not. Throughout this review, perceived stress refers to the appraisal of demands as taxing or overwhelming, whereas psychological distress refers to broader emotional symptoms or impaired functioning. Burnout denotes a sustained study-related syndrome rather than transient stress; resilience denotes adaptive capacity, coping denotes strategies used to manage demands, and well-being denotes positive psychological functioning. These constructs and their measures were reported separately and were not treated as interchangeable.

The descriptive evidence is extensive. Systematic reviews identified examinations, workload, clinical transition, and faculty or organizational factors as recurrent stressors (5, 6, 10). More recent multicenter and meta-analytic work confirms continuing psychological morbidity and substantial heterogeneity between settings (1–4). Students themselves ask for practical coping skills, credible counseling and referral pathways, constructive feedback, and changes to scheduling and learning climate (11, 12). FDI and WHO guidance likewise frame mental health as an institutional responsibility, not solely an individual capacity (13, 14).

Intervention evidence is less settled. Earlier dental-specific synthesis included only a small number of heterogeneous studies (10), while reviews across health-professions education report small or uncertain effects for resilience and mindfulness programs (15–17). Dentistry-specific evidence has expanded to include curriculum-embedded resilience modules, counseling, life coaching, meditation, app-based mindfulness, yoga, auditory interventions, cognitive reappraisal, and a direct burnout outcome. A current synthesis is therefore needed to distinguish encouraging within-group change from controlled evidence and to identify what remains untested.

This review asked: (i) which interventions have been evaluated in dental and oral health students; (ii) what effects have been reported for validated perceived-stress, psychological-distress, burnout, and resilience outcomes; and (iii) how much confidence should educators place in those findings when making curricular decisions?.

## 2 Materials and methods

### 2.1 Study design and reporting

This systematic review was conducted and reported in accordance with the PRISMA 2020 statement (18), and the protocol was registered prospectively with PROSPERO (CRD420261449695). Because clinical, methodological and outcome heterogeneity was anticipated, synthesis followed the Synthesis Without Meta-analysis (SWiM) reporting guideline (19).

Protocol adherence and deviations were assessed against the registered plan. Three post-registration deviations are reported. First, one mixed health-professions trial was retained although dentistry-specific data were not separable, because it included dental students and was the largest randomized study; it was treated as indirect, non-dental-only evidence. Second, the planned revised JBI tool for uncontrolled quasi-experimental studies was replaced by design-specific criteria adapted from the NHLBI before–after tool to address examination-cycle timing, baseline selection, maturation, regression to the mean, and attrition. Third, because studies were too sparse for separate certainty bodies by intervention category and design, GRADE was summarized by outcome; controlled evidence was prioritized for causal interpretation and uncontrolled evidence was treated as supportive or hypothesis-generating. The prespecified use of SWiM when meta-analysis was not defensible was followed and was not a deviation.

### 2.2 Eligibility criteria

Eligible populations were undergraduate or pre-registration students in dentistry, dental hygiene, dental therapy, or oral health programs. Mixed health-professions cohorts were retained when dental students were included and the intervention addressed a shared pre-registration educational context; when dental results were not separable, this was treated as indirect evidence. Eligible interventions explicitly aimed to reduce stress, distress, anxiety, depression, or burnout, or to strengthen resilience or coping. Comparators could be active control, usual support, waitlist, non-participant cohort, within-participant no-intervention periods, or none. Studies had to report at least one post-intervention outcome using a named psychometric or physiological measure. Randomized trials, non-randomized controlled studies, crossover evaluations, uncontrolled pre–post studies, and structured service evaluations with repeated outcome measurement were eligible. Cross-sectional prevalence studies, qualitative studies without an intervention, protocols, and conference abstracts were excluded.

### 2.3 Information sources and search

MEDLINE/PubMed, Embase, Scopus, Web of Science Core Collection, the Cochrane Central Register of Controlled Trials (CENTRAL), ERIC, and PsycINFO were searched for reports published from January 1, 2015, through June 30, 2026. The January 2015 start date was specified to update the earlier dental-specific syntheses (5, 6, 10, 20) and to focus on contemporary educational, digital, and curriculum-embedded interventions; the implications of this window are considered in Section 4.7. Search blocks combined dental/oral-health students, stress/burnout/resilience-related constructs, and intervention terms. ClinicalTrials.gov and the WHO International Clinical Trials Registry Platform were also searched, and reference lists of eligible reports and earlier reviews (10, 15–17) were checked. Complete source-specific strategies, including the platform, final search date, limits, and yield for each database and registry, are reproduced in Appendix S1.

### 2.4 Study selection and data extraction

Two reviewers independently screened records and full texts, with disagreements resolved by discussion and, when needed, a third reviewer. Study identities and citation details were cross-checked against the final included reports and publisher records. Extracted fields included setting, design, sample and retention, intervention content and dose, comparator, outcome instruments and timing, numerical findings, and funding. Study sample sizes were summed descriptively, but outcome-specific denominators were retained because attrition and incomplete measurement were common. No pooled effect estimate was calculated. Instead, synthesis was structured by intervention category in accordance with SWiM: for each study we tabulated the direction of effect, the reported magnitude (standardized indices such as partial *η*^2^ where available), the sample size and design tier, and 95% confidence intervals where these were reported or derivable. Because most reports omitted interval estimates, precision was appraised through sample size and is reflected in the GRADE imprecision judgments; statistical significance was recorded descriptively but was not the basis of synthesis conclusions.

### 2.5 Risk of bias and certainty of evidence

Randomized trials and studies reporting random group allocation were assessed with RoB 2 (21). Non-randomized controlled studies were assessed with ROBINS-I (22). The two crossover evaluations (23, 24) were likewise assessed with ROBINS-I because neither used randomized sequence allocation, which the crossover variant of RoB 2 presupposes; as ROBINS-I contains no crossover-specific domains, order, period, carryover washout, time-varying stressors, and paired or repeated-measures analysis were assessed explicitly and recorded under the confounding and deviation domains (Supplementary Tables 2.2, 2.4). Uncontrolled pre–post and service evaluations were assessed using criteria covering temporal confounding, maturation, regression to the mean, missing outcome data, unblinded self-report, and selective reporting. These criteria were adapted from the NHLBI Quality Assessment Tool for Before–After (Pre–Post) Studies With No Control Group (25) and supplemented by two review-specific items on examination-cycle timing and baseline selection; study-level summary judgments appear in Supplementary Table 2.3. Certainty for perceived stress/psychological distress, burnout, and resilience was rated with GRADE (26). In accordance with the deviation described in Section 2.1, one outcome-level certainty judgment was made per construct: randomized or controlled evidence anchored causal interpretation, and uncontrolled evidence was treated as supportive rather than as equivalent causal evidence.

## 3 Results

### 3.1 Study selection

**Figure 1:**
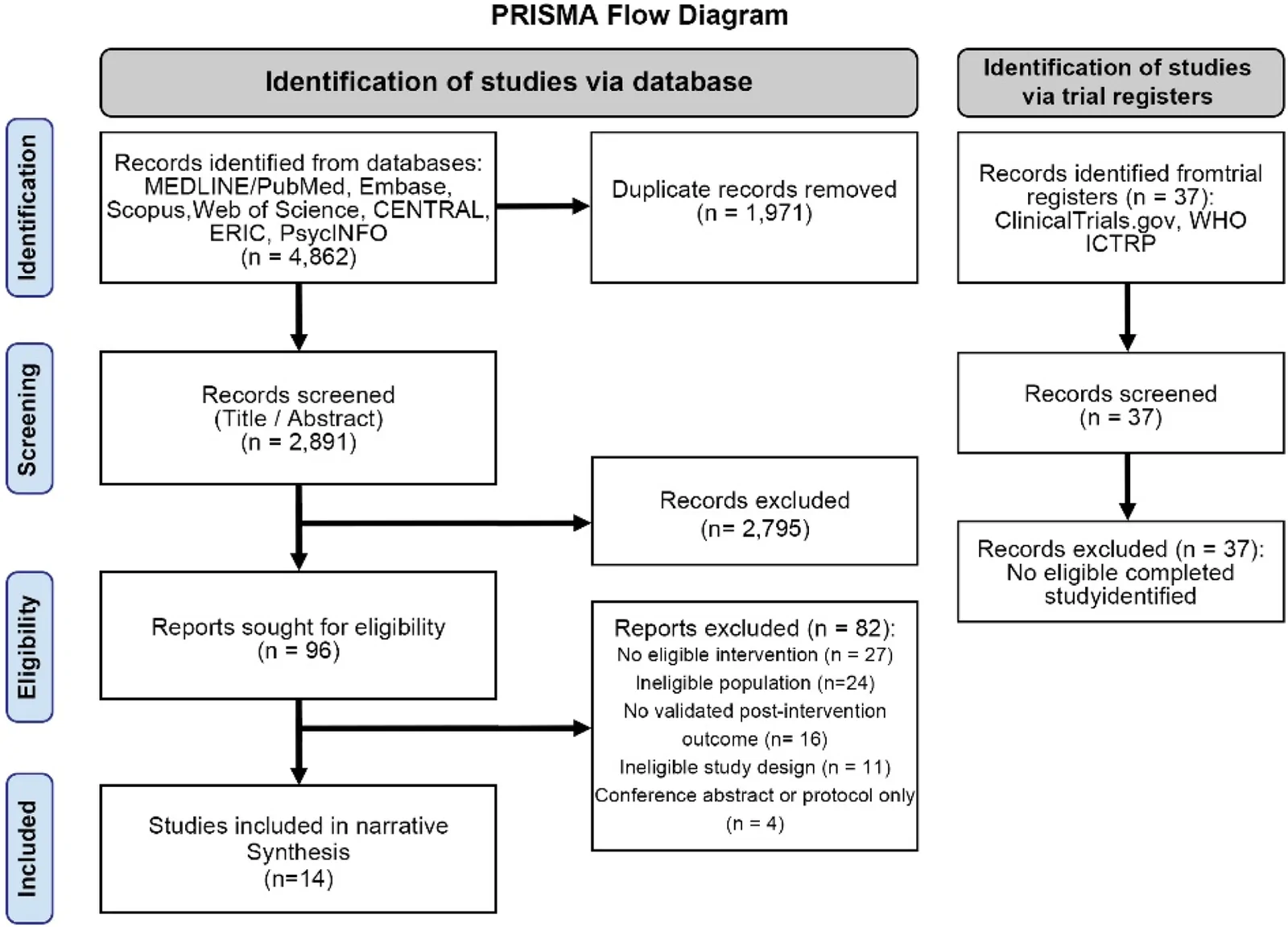
PRISMA 2020 (18) flow diagram of study identification, screening and inclusion. Records identified from databases (*n* = 4,862) and from trial registers (*n* = 37) are shown in separate identification arms.

The screening log recorded 4,862 database records, 1,971 duplicates, 2,891 records screened, and 96 full texts assessed. Register searches of ClinicalTrials.gov and the WHO ICTRP returned a further 37 records, none of which identified an additional eligible completed study. Fourteen reports met the final eligibility criteria (Figure 1). The 82 full-text exclusions were recorded as no eligible intervention (*n* = 27), ineligible population (*n* = 24), no validated post-intervention outcome (*n* = 16), ineligible design (*n* = 11), or conference abstract/protocol only (*n* = 4).

### 3.2 Characteristics of included studies

The 14 studies were conducted in 11 countries: Ireland, Germany, Thailand, Saudi Arabia (two studies), the United States (two), Finland (two), South Africa, Chile, Indonesia, Pakistan, and New Zealand (23, 24, 27–38). Reported study sample sizes totaled 750 participants; the median was 47.5 (range 5–120). This total includes 102 participants from a mixed health-professions trial in which dentistry-specific outcomes were not separable (37) and 32 dental-hygiene students (36). Excluding the mixed cohort, the dental- and oral-health-specific total was 648 participants; this caveat applies wherever the 750-participant total is cited. Sex reporting was incomplete, and three studies enrolled single-sex samples: two all-female cohorts (27, 36) and one all-male cohort (23).

Three studies were explicitly randomized controlled trials (36–38). Hayati et al. reported random assignment to three groups but described the study as quasi-experimental and did not provide enough detail to verify the randomization process (35). Four additional studies used non-randomized comparison or within-participant crossover designs (23, 24, 27, 28), and six were uncontrolled pre–post, service, or case-series evaluations (29–34). Formal fixed follow ranged from immediate post-session measurement to six months; one embedded-service evaluation reported repeated contacts across three semesters but did not use a uniform endpoint (29). Table 1 presents study-level details.

**Table 1:** Characteristics and principal findings of the 14 included studies.

Study; country	Design; sample	Intervention; comparator	Outcomes; timing	Principal finding
Aboalshamat et al. (2020) (27); Saudi Arabia	Non-randomized controlled interventional study; 88 analyzed (44 intervention; 44 control); 97 began	One introductory lecture plus five weekly 15-min telephone life-coaching sessions using the GROW model Comparator: No coaching	DASS-21, RS-14, perceived wellness, goal approach; pre/post Follow-up: Immediately after five weeks	Between-group time effects favored coaching for depression (*p* = 0.026, *ηp*^2^ = 0.026) and stress (*p* = 0.010, *ηp*^2^ = 0.074); anxiety and resilience did not improve vs. control.
Alzahem et al. (2015) (23); Saudi Arabia	Non-randomized waitlist crossover; 31 (15 third-year; 16 s-year); all male	Dental Education Stress Management program: three 90-min sessions over three weeks (psychoeducation, breathing, CBT, time management) Comparator: Staggered waitlist cohort	DES and PSM-9 at three time points Follow-up: Two weeks after each cohort completed the program	Group-time interactions favored the program for DES [F(2,58) = 10.36, *ηp*^2^ = 0.22] and PSM-9 [F(2,58) = 8.19, *ηp*^2^ = 0.04]; effects persisted for at least two weeks.
Felszeghy et al. (2024) (24); Finland	Within-participant crossover evaluation; 31 (24 female; 7 male)	Self-selected background music and deep belly breathing during fixed-prosthodontic simulation exercises Comparator: Practice sessions without the coping strategies	GHQ-12, PHQ-4, and VAS-Anxiety Follow-up: End of preclinical course/immediate exercise ratings	GHQ-12 stress decreased in women (*p* = 0.025) but not men; music and breathing reduced VAS fear/stress, although a clear contrast with non-use was not demonstrated.
Lin et al. (2026) (28); Germany	Non-randomized controlled pre–post study; 56 (27 training; 29 non-participant controls)	Two-day online resilience training tailored to dental-school stressors Comparator: Non-participant student controls	Resilience, perceived stress, life satisfaction, health behavior; online surveys Follow-up: Six-month study window	Medium group-time effect on resilience [F(1,54) = 4.93, *p* = 0.031, η^2^ = 0.084]; lower subjective stress and higher life satisfaction were reported; health-behavior findings were inconclusive.
Adams (2017) (29); United States	Uncontrolled embedded-service evaluation; 55 students; 251 appointments	Individual counseling embedded within the dental school; mean 4.5 appointments/student Comparator: None	CCAPS-34 and Outcome Rating Scale across counseling contacts Follow-up: Repeated contacts over three semesters; no uniform endpoint	At intake, 45/55 (82%) had a clinically significant score on at least one CCAPS-34 subscale; more appointments were associated with better overall functioning, without a causal comparator.
Anishchuk et al. (2026) (30); Ireland	Uncontrolled curriculum-embedded pre–post evaluation; 62 baseline respondents; post-test denominator not clearly reported	ResiliDents asynchronous eLearning module on resilience and coping; embedded in curriculum Comparator: None	CD-RISC-25 before and after the module Follow-up: Immediate post-module	CD-RISC-25 63.84 (SD 15.69) to 68.87 (SD 18.06), +7.9%, not statistically significant; coping subscale improved (*p* < 0.01).
Fourie et al. (2026) (31); South Africa	Uncontrolled prospective pilot; 31 enrolled; 26 baseline; 9 follow-up	Medito smartphone app, 10 min/day for eight weeks Comparator: None	PSS-10, 0–10 self-rated stress, app use Follow-up: Eight weeks	PSS-10 25.81 (SD 7.96) to 20.33 (SD 8.31), *p* = 0.50; self-rated stress 7.54 to 5.78, *p* = 0.50. Mean use was only 53 min over about six days.
González and Quezada (2016) (32); Chile	Uncontrolled A-B-C multiple-case series; 5	Eight-session individual cognitive-behavioral intervention Comparator: None	OQ-45.2, Dental Environment Stress questionnaire, exit interview Follow-up: One month after the last session	All five reported lower dental-environment stress; two participants moved from dysfunctional to normal OQ-45.2 scores; the remaining three stayed in the normal range.
Islam et al. (2026) (33); Pakistan	Uncontrolled mixed-methods quasi-experimental study; 69 (37 female; 32 male)	Two 2-h mindfulness/life-skill-building sessions delivered on one day Comparator: None	CBI-SS before and immediately after; stakeholder focus groups Follow-up: Immediate post-session	Personal burnout (mean difference 1.62, *p* = 0.003) and study-related burnout (1.88, *p* = 0.005) decreased; colleague- and teacher-related domains did not change significantly.
Patarathipakorn et al. (2025) (34); Thailand	Uncontrolled quasi-experimental study; 31 selected; 28 completed	Anapanasati meditation: four workshops plus 60 min/day walking and sitting meditation for 14 weeks Comparator: None	T-PSS-10 at baseline, week 14, and week 18; group discussion Follow-up: Four weeks after intervention	T-PSS-10 17.7 to 9.8 to 8.4 (*p* < 0.001). Large within-person changes cannot be separated from time, selection, or examination-cycle effects.
Hayati et al. (2026) (35); Indonesia	Three-arm quasi-experimental study reporting random group allocation; 120	40-Hz isochronic tones for 30 min/day or 10 min/day for 14 days Comparator: No-tone control	DASS before and after 14 days Follow-up: Immediate post-intervention	The 30-min group improved within group for anxiety, depression, and stress (all *p* < 0.05); post-intervention group differences were reported for anxiety and depression, not stress.
Kanderis Lane et al. (2021) (36); United States	Non-blinded randomized controlled trial; 32 (16 yoga; 16 control); all female	Six 15-min video-guided yoga/breathing sessions immediately before final examinations Comparator: Usual pre-examination routine	PSS-10, blood pressure, pulse Follow-up: End of finals week	PSS fell within the yoga group by 1.8 points (*p* < 0.001), but post-trial PSS did not differ from control (*p* = 0.68); several acute physiological measures improved.
Repo et al. (2022) (37); Finland	Three-arm randomized controlled trial; 102 (40 face-to-face; 22 web ACT; 40 control)	Mindfulness Skills for Students course or web-based Student Compass ACT program Comparator: Student mental-health support as usual	CORE-OM primary; stress, well-being, mindfulness, hair cortisol secondary Follow-up: Post-intervention and four months	Primary CORE-OM differences were not significant (face-to-face *p* = 0.076; web *p* = 0.516; pooled *p* = 0.067); functioning/social interaction favored face-to-face and pooled groups (p = 0.028). No primary group difference remained at four months.
Smith et al. (2019) (38); New Zealand	Randomized active-control trial; 40 baseline respondents (20/group) from 84 invited	4.5-min video teaching cognitive reappraisal Comparator: 4.5-min generic stress-management video	Modified 20-item Dental Environment Stress questionnaire Follow-up: Reported as three weeks; methods also state surveys were eight weeks apart	Mean DES change favored reappraisal (+3.10 vs. −1.06), but the between-group difference was not statistically significant (*p* = 0.076).

Statistical symbols: F, F statistic; *p*, probability value; η^2^, eta squared; ηp^2^, partial eta squared.

A–B–C, baseline–intervention–follow-up phase design; ACT, acceptance and commitment therapy; CBI-SS, Copenhagen Burnout Inventory–Student Survey; CCAPS-34, 34-item Counseling Center Assessment of Psychological Symptoms; CBT, cognitive behavioral therapy; CD-RISC-25, 25-item Connor–Davidson Resilience Scale; CORE-OM, Clinical Outcomes in Routine Evaluation–Outcome Measure; DASS/DASS-21, Depression Anxiety Stress Scales/21-item version; DES, Dental Environment Stress questionnaire; GHQ-12, 12-item General Health Questionnaire; GROW, Goal, Reality, Options, and Will; OQ-45.2, Outcome Questionnaire-45.2; PHQ-4, 4-item Patient Health Questionnaire; PSM-9, 9-item Psychological Stress Measure; PSS/PSS-10, Perceived Stress Scale/10-item version; RS-14, 14-item Resilience Scale; SD, standard deviation; T-PSS-10, Thai version of the 10-item Perceived Stress Scale; VAS, visual analog scale.

### 3.3 Effectiveness by intervention category

**Figure 2:**
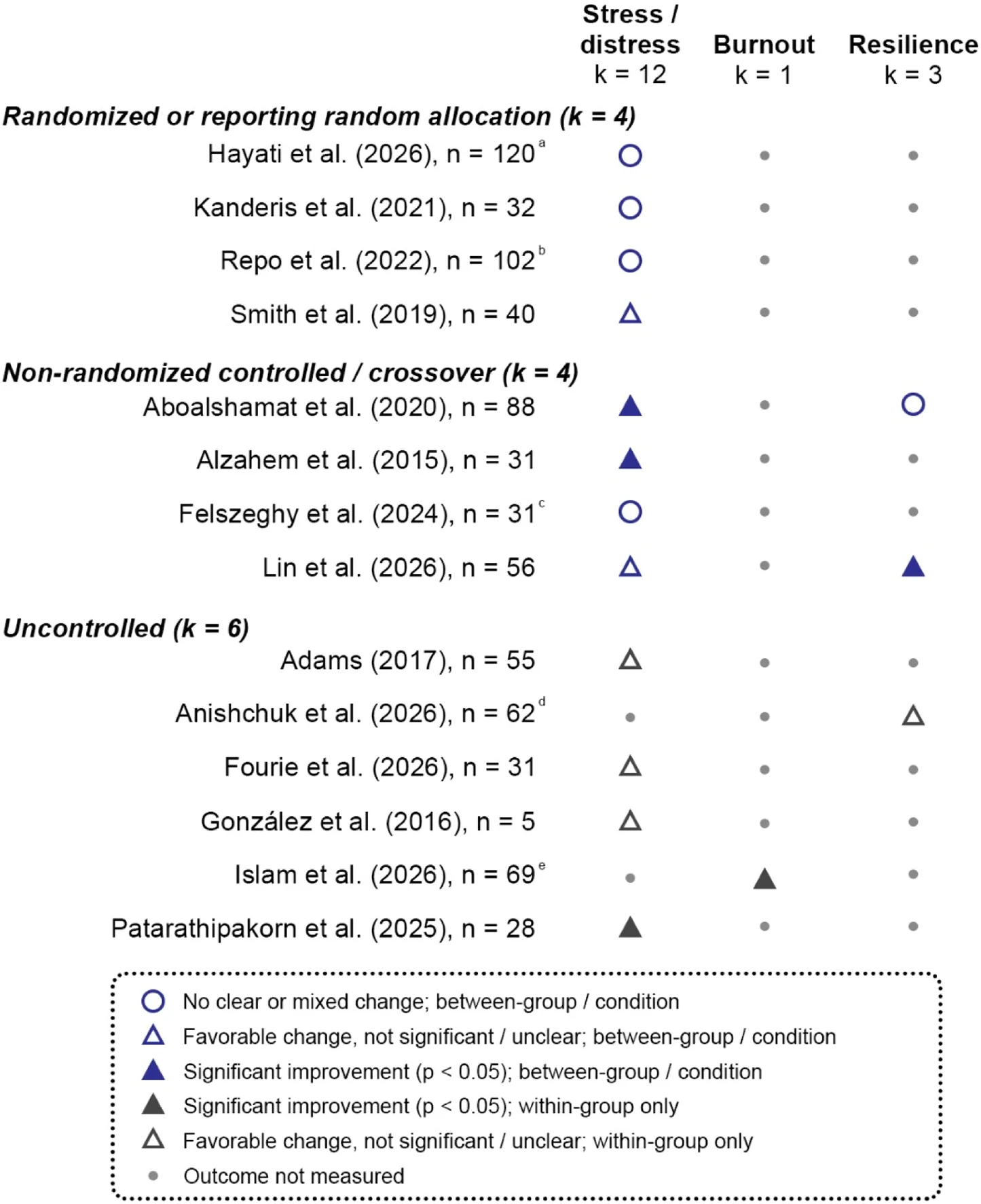
Study-design-stratified evidence map for validated perceived-stress/psychological-distress, burnout, and resilience outcomes. Each symbol represents the direction of the principal validated outcome. For controlled or crossover studies, between-group or between-condition evidence was prioritized over within-group change; uncontrolled studies were coded using within-group evidence only. Filled symbols indicate *p* < 0.05 for the comparison represented; open symbols indicate a non-significant result or insufficiently reported statistical significance. *n* denotes the reported study sample size used in this review; outcome-specific denominators were sometimes smaller because of attrition. The figure is descriptive and does not represent effect magnitude or certainty of evidence. ᵃ Random allocation reported within a quasi-experimental design; between-group differences favored anxiety and depression but not stress. ᵇ Primary CORE-OM comparisons were not significant; a functioning/social-interaction subdomain favored face-to-face delivery. ᶜ Sex-specific within-participant improvement (women only); a clear contrast with non-use was not demonstrated. ᵈ Total CD-RISC-25 change was not significant; the coping subscale improved. ᵉ Personal and study-related burnout improved; colleague- and teacher-related domains were unchanged.

Twelve studies reported perceived stress or broader psychological distress, three reported resilience, and one directly measured burnout. Instruments varied substantially (Table 2). PSS-10 was used in several meditation, yoga, and digital-intervention studies; other studies used dental-environment stress scales, DASS, CORE-OM, GHQ-12, CCAPS-34, OQ-45.2, CD-RISC-25, RS-14, or the Copenhagen Burnout Inventory–Student Survey. Physiological measures appeared in two randomized studies but did not provide consistent comparative evidence (36, 37). Figure 2 provides a study-design-stratified evidence map that distinguishes randomized or reported-random-allocation studies, non-randomized controlled/crossover studies, and uncontrolled studies. Its symbols are descriptive; the synthesis itself weighed direction, magnitude, sample size, precision, follow-up, and design tier rather than statistical significance alone (Section 2.4).

**Table 2:** Principal outcome instruments represented in the included studies.

Instrument	Construct measured	Included applications	Interpretive issue
PSS-10/T-PSS−10 (39)	Perceived stress over the previous month	Meditation, app, and yoga studies (31, 34, 36)	Sensitive to examination timing; scores are not burnout measures.
DASS/DASS-21 (40)	Depression, anxiety, and stress	Life coaching and isochronic tones (27, 35)	Subscales should be interpreted separately.
CD-RISC-25/RS-14 (41)	Resilience or adaptive capacity	ResiliDents, German training, and life coaching (27, 28, 30)	Short interventions may change coping without changing total resilience.
CBI-SS	Personal, study-, colleague-, and teacher-related burnout	Mindfulness life-skill sessions (33)	Only direct burnout endpoint in the included set; immediate uncontrolled measurement.
CORE-OM	Psychological distress, functioning, symptoms, well-being, risk	Mixed health-professions mindfulness/ACT RCT (37)	Dental-specific effects were not separable.
DES/PSM-9	Dental-environment or general psychological stress	Stress-management program, CBT case series, cognitive reappraisal (23, 32, 38)	Modified versions and scoring limit cross-study comparability.
CCAPS-34/ORS/OQ-45.2/GHQ-12/PHQ-4	Clinical distress, functioning, common symptoms	Counseling, case series, music/breathing (24, 29, 32)	Useful for services, but exposure and follow-up were not standardized.

ACT, acceptance and commitment therapy; CBI-SS, Copenhagen Burnout Inventory–Student Survey; CCAPS-34, 34-item Counseling Center Assessment of Psychological Symptoms; CBT, cognitive behavioral therapy; CD-RISC-25, 25-item Connor–Davidson Resilience Scale; CORE-OM, Clinical Outcomes in Routine Evaluation–Outcome Measure; DASS/DASS-21, Depression Anxiety Stress Scales/21-item version; DES, Dental Environment Stress questionnaire; GHQ-12, 12-item General Health Questionnaire; OQ-45.2, Outcome Questionnaire-45.2; ORS, Outcome Rating Scale; PHQ-4, 4-item Patient Health Questionnaire; PSM-9, 9-item Psychological Stress Measure; PSS-10, 10-item Perceived Stress Scale; RCT, randomized controlled trial; RS-14, 14-item Resilience Scale; T-PSS-10, Thai version of the 10-item Perceived Stress Scale.

**Mindfulness and meditation (four studies; *n* = 230).** The Thai 14-week meditation study reported a large uncontrolled PSS reduction that persisted four weeks after the program (34). Without a comparator, however, this change cannot be attributed to the meditation program with confidence: baseline selection, examination timing, and adaptation across the first year offer competing explanations (Section 4.2). The South African app pilot also showed numerically lower PSS, but only 9 of 26 baseline participants completed follow-up and mean use was 53 min across eight weeks (31). In an uncontrolled same-day evaluation, two mindfulness sessions were followed by lower personal and study-related burnout scores in 69 final-year students, while colleague- and teacher-related burnout did not change (33). In the mixed health-professions RCT, primary CORE-OM differences did not reach statistical significance and no group difference remained at four months (37).

**Coaching, counseling, cognitive-behavioral, and psychoeducational approaches (five studies; *n* = 219).** Life coaching improved stress and depression relative to a non-randomized control group, but not anxiety or resilience (27). A staggered waitlist study reported improvements in dental and general stress after a three-session program (23). Embedded counseling reached students with substantial clinical distress, but dose was determined by service use and there was no untreated comparator (29). All five participants in a CBT case series improved on dental-environment stress (32). A randomized cognitive-reappraisal video produced a favorable but non-significant between-group change (*p* = 0.076) (38).

**Movement, sensory, and relaxation approaches (three studies; *n* = 183).** Six brief yoga sessions during finals week reduced PSS within the yoga group, but post-trial PSS did not differ from control (36). In a crossover preclinical-course evaluation, music and deep breathing reduced immediate VAS fear/stress and GHQ-12 improved among women, yet a clear contrast with no coping strategy was not shown (24). A 14-day isochronic-tone study reported within-group changes in all DASS domains for the 30-minute condition and between-group differences for anxiety and depression, but not stress (35).

**Resilience training (two studies; *n* = 118).** The German non-randomized study found a medium group-time effect on resilience and reported lower subjective stress over a six-month window (28). ResiliDents increased mean CD-RISC-25 by 7.9% without statistical significance; only the coping subscale improved (30). These programs support feasibility and proximal skill development more clearly than durable global resilience. No eligible study evaluated an organization-level intervention that modified assessment density, scheduling, clinical quotas, or learning climate. Table 3 consolidates the category-level synthesis, including this evidence gap.

**Table 3:** Category-level synthesis of the included evidence. Participant counts include the mixed health-professions cohort (*n* = 102) within the mindfulness/meditation category (Section 3.2).

Category	Studies; participants	Pattern of findings	Interpretation
Mindfulness/meditation	4; 230	Large uncontrolled changes in two studies; high attrition in one app pilot; no clear primary effect or persistence in the mixed-cohort RCT	Very uncertain; adherence and facilitator contact appear important but are confounded with design.
Coaching/counseling/CBT/psychoeducation	5; 219	Some favorable controlled and service outcomes; randomized cognitive-reappraisal result not significant; very small case series	Potentially useful access pathway, but effectiveness and prevention should not be conflated.
Movement/sensory/relaxation	3; 183	Acute or within-group improvements; comparative stress findings were absent or non-significant	Suitable for pragmatic testing around high-arousal events; durability unknown.
Resilience training	2; 118	One medium non-randomized effect; one non-significant total-score change with coping-subscale improvement	Feasible, including curriculum-embedded delivery; causal certainty remains low.
Organization-level prevention	0	No eligible intervention changed assessment density, scheduling, quotas, or learning climate	Critical evidence gap; individual programs do not test reduction of stressor exposure.

CBT, cognitive behavioral therapy; RCT, randomized controlled trial.

### 3.4 Risk of bias and certainty

**Figure 3:**
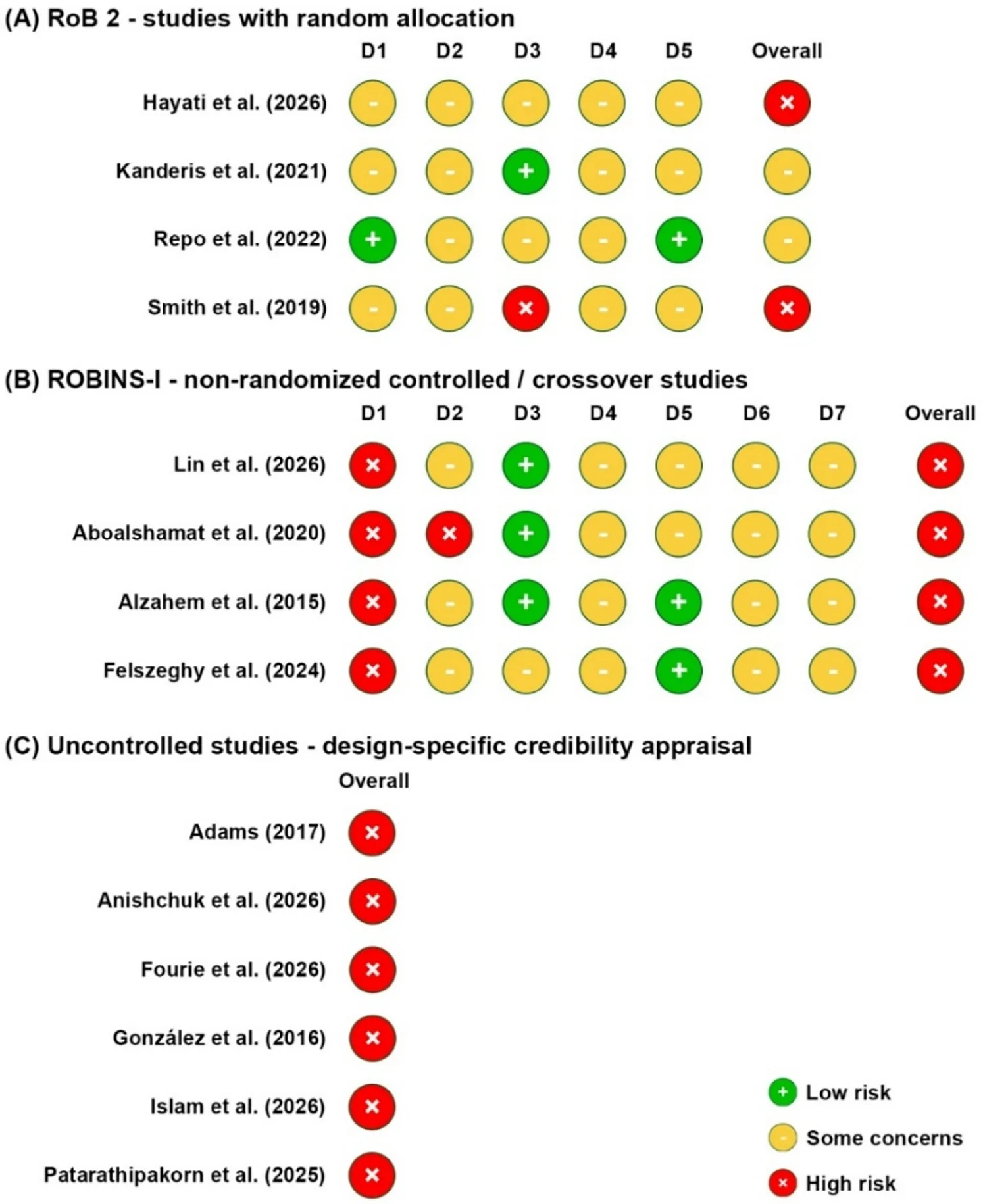
Risk of bias in the included studies. **(A)** RoB 2 judgments for the four studies with random allocation: D1 randomization process; D2 deviations from intended interventions; D3 missing outcome data; D4 measurement of the outcome; D5 selection of the reported result. **(B)** ROBINS-I judgments for the four non-randomized controlled or crossover studies: D1 confounding; D2 participant selection; D3 classification of interventions; D4 deviations from intended interventions; D5 missing data; D6 outcome measurement; D7 selection of the reported result. **(C)** Overall credibility appraisal of the six uncontrolled studies against pre-specified design-specific criteria. Judgments refer to each study's principal self-reported outcome and correspond to Supplementary Tables 2.1–S2.3.

No study provided low-risk evidence for its principal self-reported outcome. Among studies with random allocation, two raised some concerns and two were judged high risk, driven by unclear allocation methods, lack of blinding, missing outcome data, or selective-reporting concerns (35–38). All four non-randomized comparison/crossover studies were at serious risk of bias, principally from confounding, self-selection, time or order effects, and unblinded self-report (23, 24, 27, 28). The six uncontrolled studies had serious credibility limitations; particularly important were same-day outcome measurement (33), 65% follow-up loss (31), baseline selection (34), a five-person case series (32), and non-standardized counseling exposure (29). Figure 3 presents the domain-level judgments; the full assessment tables are in Appendix S2.

GRADE certainty was very low for all three outcomes, and the downgrading pathway differed by outcome (Table 4). For perceived stress/psychological distress, the evidence was downgraded for very serious risk of bias (no study contributed low-risk evidence, and several influential studies had high or serious risk; −2), serious inconsistency between frequent within-group improvement and mixed or non-significant controlled comparisons (−1), serious indirectness because the largest randomized trial enrolled a mixed health-professions cohort (−1) (37), and serious imprecision arising from small samples with largely unreported confidence intervals (−1). For burnout, the single uncontrolled study (33) started at low certainty and was downgraded for serious risk of bias (−1) and serious imprecision because it provided immediate-only measurement without a comparator or confidence interval (−1). For resilience, the non-randomized evidence started at low certainty and was downgraded for serious risk of bias (−1), inconsistency between the medium controlled effect (28), the non-significant total-score change (30), and the null coaching comparison(−1) (27), and imprecision (−1).

**Table 4:** GRADE summary of findings: interventions to reduce stress or burnout, or to build resilience, vs. any comparator in undergraduate dental students. GRADE, Grading of Recommendations Assessment, Development and Evaluation; certainty ratings: ⊕⊕⊕⊕ high, ⊕⊕⊕⊙ moderate, ⊕⊕⊙⊙ low, ⊕⊙⊙⊙ very low. Participant totals include the mixed health-professions cohort where applicable (Section 3.2).

Outcome	Studies; participants	Summary	Certainty (GRADE)	Reasons
Perceived stress/psychological distress	12; 619	Within-group improvement was common; controlled primary effects were mixed or non-significant, and longer-term effects were generally absent	Very low ⊕⊙⊙⊙	Risk of bias, inconsistency, imprecision, and some indirectness
Burnout	1 uncontrolled; 69	Immediate improvement in personal and study-related domains; no significant change in colleague- or teacher-related burnout	Very low ⊕⊙⊙⊙	Serious risk of bias, single study, no comparator, no follow-up
Resilience	3; 206	One medium non-randomized group-time effect; one non-significant total CD-RISC change; coaching did not improve resilience vs. control	Very low ⊕⊙⊙⊙	Serious risk of bias, inconsistency, imprecision

CD-RISC, connor–davidson resilience scale.

## 4 Discussion

### 4.1 Principal findings

Four conclusions emerge from the 14 included studies. First, the intervention literature is broader than mindfulness and resilience training: it now includes counseling, life coaching, CBT, stress-management psychoeducation, yoga, music and breathing, auditory stimulation, and cognitive reappraisal. Second, positive within-group change is frequent but controlled evidence is far less consistent. The three explicit RCTs did not show a clear primary stress advantage at their main endpoint (36–38), and the quasi-experimental auditory study reported between-group effects for anxiety and depression rather than stress (35). Third, burnout is no longer an entirely unmeasured outcome, but the only direct study used an immediate uncontrolled pre–post design (33). Fourth, no included intervention reduced the structural stressors that students identify, such as assessment clustering, clinical scheduling, quotas, or learning climate.

### 4.2 Why the findings should not be over-read?

The largest numerical changes came from designs most vulnerable to alternative explanations. The Thai meditation cohort was selected using baseline stress, lacked a comparator, and was followed across a semester (34). Regression to the mean, adaptation to first-year demands, and the examination calendar could therefore contribute to the observed decline. The app pilot illustrates the opposite problem: a potentially scalable intervention had only nine follow-up completers and minimal use (31). In both cases, the point estimate alone says little about what would happen under routine implementation.

### 4.3 Intervention heterogeneity, construct, and timing

**Figure 4:**
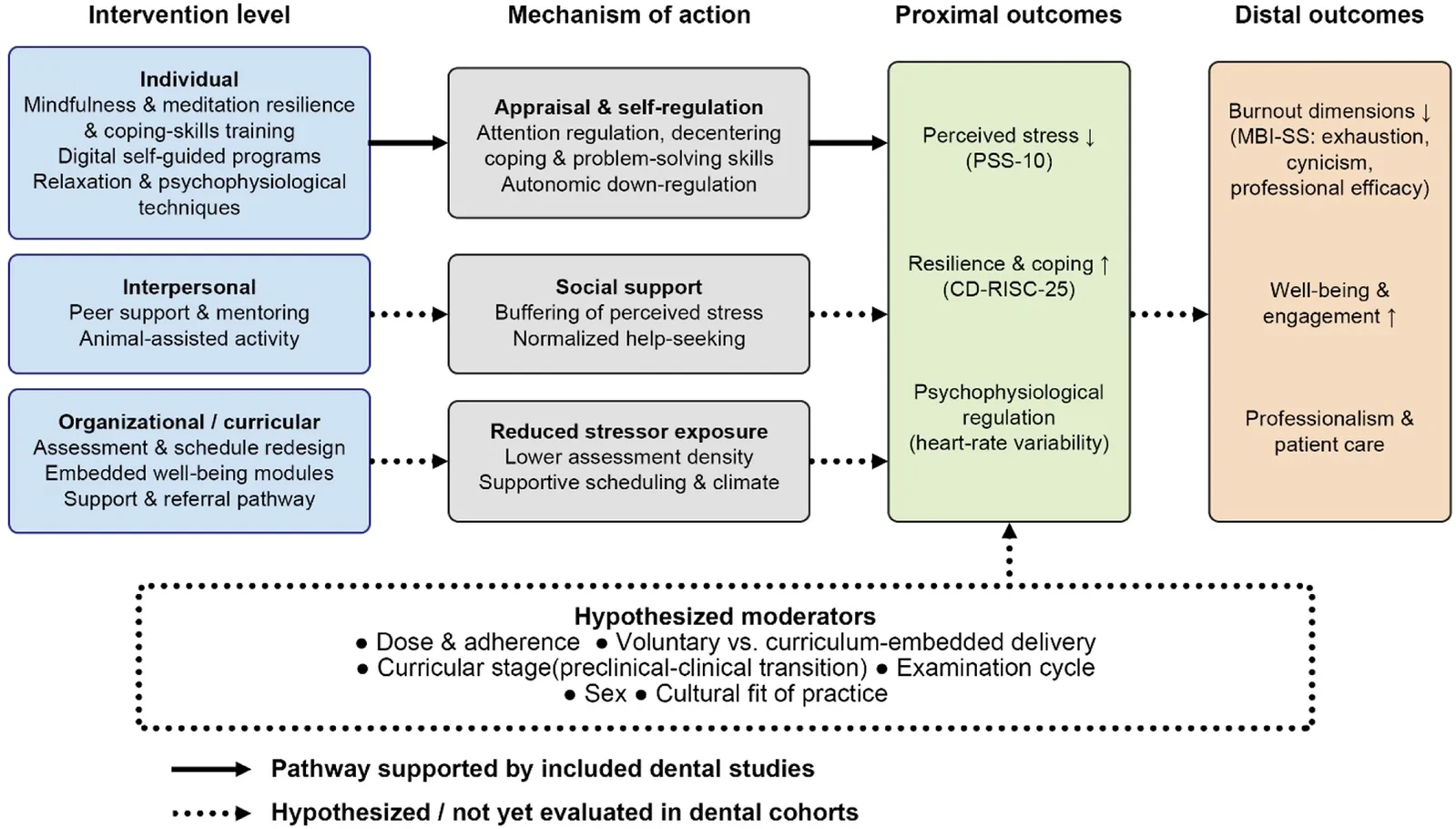
Conceptual framework linking intervention level and theoretical mechanism to proximal and distal outcomes. Solid pathways denote domains represented in the included dental/oral-health studies; dotted pathways remain hypothesized or incompletely evaluated. The lower panel identifies mechanism–target fit, dose/intensity and adherence, delivery support, curricular stage, examination timing, embedding, and cultural fit as potential modifiers of effectiveness.

Intervention effects can be interpreted through five linked characteristics: theoretical target and mechanism; duration, intensity, and total dose; delivery mode and human support; timing within the curriculum or examination cycle; and alignment between the intended mechanism, outcome, and follow-up. Greater dose and rehearsal may be needed for skills or resilience; facilitator contact may support engagement; self-guided delivery trades reach for adherence; and measurement during an assessment peak may detect transient arousal rather than durable change. Figure 4 places these characteristics within a multilevel program logic and identifies them as potential effect modifiers. Because included studies combined these features differently, favorable and null findings should not be treated as estimates of one common intervention effect.

Perceived stress, psychological distress, coping, resilience, well-being, and burnout occupy different positions in the causal chain. A short exercise can reduce momentary arousal, a skills course can alter coping, and counseling can improve functioning; none of these outcomes proves that sustained burnout has been prevented. Islam et al. provide direct burnout data, but measurement immediately after two sessions cannot establish durability (33). Similarly, the total resilience score changed little after ResiliDents even though coping improved (30), which is plausible for a proximal skill after a brief module. Future evaluations should select outcomes that match the intervention mechanism and should prespecify clinically and educationally meaningful follow-up.

### 4.4 Delivery, access, and healthy-volunteer bias

Voluntary programs can preferentially attract students who have time, interest, or confidence to seek help. The German resilience comparison (28), counseling service (29), and several mindfulness studies are all susceptible to this form of selection. Curriculum-embedded delivery may improve equity of access, but compulsory participation does not guarantee practice or benefit. Repo et al. found better engagement in facilitated mindfulness than in the web program, while Fourie et al. documented very low app use (31, 37). Delivery mode should therefore be evaluated as an implementation component rather than assumed to be neutral.

### 4.5 Implications for current dental education

At present, the evidence supports feasibility more clearly than effectiveness. Dental schools may reasonably offer confidential counseling and referral pathways, brief curriculum-embedded coping or resilience education, and optional facilitated mindfulness, breathing, or movement sessions as adjunctive supports, provided access is equitable, participation is not framed as remediation, and implementation includes adherence and outcome monitoring. These choices are supported by observed deliverability and acceptability, not by high-certainty evidence of stress or burnout prevention.

Stand-alone self-guided applications without engagement support, isochronic-tone or other sensory approaches, very brief interventions claimed to produce durable resilience, and any program promoted as preventing burnout should remain experimental pending adequately controlled replication and longer follow-up. Universal or mandatory roll-out should be preceded by piloting because effects may depend on dose, facilitator contact, timing, cultural fit, and student preference.

### 4.6 Organization-level interventions: a critical evidence gap

All 14 eligible interventions primarily asked students to change their appraisal, behavior, self-regulation, or help-seeking; none evaluated an intervention designed principally to reduce exposure to organization-level stressors. This finding should not be interpreted as proof that dental schools lack institutional initiatives. Rather, it shows that curriculum- and policy-level changes have not been evaluated with the eligible designs and validated outcomes represented in this review.

Future priorities include curriculum mapping and redesign to remove avoidable overload; coordinated examination scheduling; optimization of clinical workload, requirements, and quotas; formal mentoring systems; faculty development in feedback, supervision, and psychological safety; and institutional mental-health policies that define confidential access, referral, crisis response, and reasonable accommodation. These approaches should be co-designed with students and faculty, accompanied by process measures such as assessment density, workload, reach, and fidelity, and evaluated using cluster-randomized, stepped-wedge, or controlled interrupted time-series designs. Multilevel programs should pair student support with auditable reduction of structural stressors so that responsibility is not shifted to students for conditions created by the curriculum.

### 4.7 Strengths and limitations

Strengths of this review include coverage of the 14 reports identified within the 2015–2026 search window, study-level reconciliation of sample and outcome denominators, design-specific risk-of-bias assessment, and explicit separation of direct dental evidence from one mixed health-professions trial (37). Limitations remain. Several reports provided incomplete denominators or inconsistent timing, and one report contained internal inconsistencies in study dates (34). Clinical and methodological heterogeneity precluded meta-analysis. Quantitative assessment of publication bias using funnel plots or regression tests was not feasible because no sufficiently comparable outcome–intervention group contained enough controlled studies, effect estimates and variances were frequently unavailable, and designs, measures, and time points differed substantially. Registry searches reduced but did not eliminate concern; selective publication and selective outcome reporting may therefore have inflated favorable findings. The mixed cohort increases indirectness, although excluding it would remove the largest randomized mindfulness trial containing dental students. Most remaining studies were small, single-center, unblinded, and vulnerable to self-selection or examination-cycle effects. In addition, the January 2015 start date means the review characterizes the contemporary decade of intervention research rather than the complete historical record; earlier studies were covered by previous syntheses (5, 6, 10).

### 4.8 Research priorities

Priority studies are multicenter randomized or stepped-wedge evaluations with concealed allocation where feasible, a prespecified primary outcome, and follow-up of at least one academic year. Burnout trials should report validated domains rather than a single composite, and stress trials should record examination timing. Digital programs should report objective engagement and analyze attrition. Organizational interventions require designs capable of evaluating policy or curriculum change, such as interrupted time series or stepped-wedge implementation. A core outcome set for oral-health education would reduce the present fragmentation of instruments.

## 5 Conclusions

The 14 included studies do not justify a claim that any single intervention reliably prevents perceived stress or burnout in dental and oral health students. They support offering selected student-facing services as carefully evaluated adjuncts, particularly confidential counseling/referral and brief supported skills programs, but they do not establish durable effectiveness. Stand-alone digital or sensory interventions and claims of burnout prevention should remain experimental. The effects are inconsistent across controlled studies, direct burnout evidence is limited to one immediate uncontrolled evaluation, and long-term and organization-level evidence is absent. Dental schools should therefore pair student support with rigorous evaluation and action on curricular and institutional sources of stress.

## Data Availability

The original contributions presented in the study are included in the article/Supplementary Material, further inquiries can be directed to the corresponding author/s.
