# Supplementary material for: Interventions for stress, burnout, and resilience in dental and oral health students: a systematic review
Source: Front Med (Lausanne). 2026 Sep 7;13:1945728. doi: 10.3389/fmed.2026.1945728 (PMC13590300; doi:10.3389/fmed.2026.1945728)
Supplement: Supplementary file 1 [file Supplementaryfile1.docx]

**Supplementary Material**

**Interventions for Stress, Burnout, and Resilience in Dental and Oral Health Students: A Systematic Review**

**Author**

Xiaohong Deng^1, 2, 3^, Wenjuan Qiang^1, 2, 3^, Tiezhou Hou^1, 2, 3 *^ and Le Qiang^1, 2, 3 *^

1. Key laboratory of Shaanxi Province for Craniofacial Precision Medicine Research, College of Stomatology, Xi’an Jiaotong University

2. Clinical Research Center of Shaanxi Province for Dental and Maxillofacial Diseases, College of Stomatology, Xi’an Jiaotong University

3. Department of Cariology and Endodontics, College of Stomatology, Xi’an Jiaotong University

Corresponding author:

Tiezhou Hou, tiezhou@mail.xjtu.edu.cn;

Le Qiang, qiangle@xjtu.edu.cn

**Contents**

Appendix S1. Information sources and full search strategies ................................................... 2

Appendix S2. Risk-of-bias assessments ……………………………………………………… 5

Appendix S3. Detailed characteristics and findings of all included studies ............................. 8

References ............................................................................................................................... 11

# Appendix S1. Information sources and full search strategies

The final search update covered publications from January 1, 2015, through June 30, 2026, with no language restriction. The lower date limit was intended to update the earlier dental-specific review and focus on contemporary educational and digital delivery contexts. Strategies were developed for MEDLINE/PubMed and adapted to each platform. Database searches yielded 4,862 records. ClinicalTrials.gov and WHO ICTRP yielded 37 registry records, which were screened separately and were not included in the database deduplication pathway; none identified an additional eligible completed study. Each source is presented separately below with its interface, date, yield, limits, and complete strategy in an accessible format.

**Table S1.1** Sources, final search date, and records in the screening log.

| **Source** | **Interface / platform** | **Date searched** | **Records** |
| --- | --- | --- | --- |
| MEDLINE | PubMed (NLM) | 30 June 2026 | 1,204 |
| Embase | Embase.com (Elsevier) | 30 June 2026 | 1,391 |
| Scopus | Scopus.com (Elsevier) | 30 June 2026 | 987 |
| Web of Science Core Collection | Clarivate | 30 June 2026 | 764 |
| CENTRAL | Cochrane Library (Wiley) | 30 June 2026 | 213 |
| ERIC | EBSCOhost | 30 June 2026 | 121 |
| PsycINFO | Ovid | 30 June 2026 | 182 |
| Total (databases) | — | — | 4,862 |
| ClinicalTrials.gov | clinicaltrials.gov | 30 June 2026 | 24 |
| WHO ICTRP | trialsearch.who.int | 30 June 2026 | 13 |

**S1.2 MEDLINE (PubMed) — executable strategy**

Platform: PubMed (NLM); searched 30 June 2026; yield: 1,204; limits: publication date 1 January 2015–30 June 2026; no language restriction.

| **Line** | **Search string** |
| --- | --- |
| #1 Population | "Students, Dental"[Mesh] OR "dental student*"[tiab] OR "dentistry student*"[tiab] OR "dental hygiene student*"[tiab] OR "dental hygienist student*"[tiab] OR "dental therapy student*"[tiab] OR "oral health student*"[tiab] OR ((dental[tiab] OR dentistry[tiab] OR "oral health"[tiab] OR stomatolog*[tiab]) AND (student*[tiab] OR undergraduate*[tiab] OR "pre-registration"[tiab])) |
| #2 Outcomes | "Stress, Psychological"[Mesh] OR "Burnout, Psychological"[Mesh] OR "Burnout, Professional"[Mesh] OR "Resilience, Psychological"[Mesh] OR stress*[tiab] OR distress*[tiab] OR burnout[tiab] OR burn-out[tiab] OR "burn out"[tiab] OR resilien*[tiab] OR wellbeing[tiab] OR "well-being"[tiab] OR "well being"[tiab] OR anxiety[tiab] OR coping[tiab] OR "mental health"[tiab] |
| #3 Intervention | "Mindfulness"[Mesh] OR "Meditation"[Mesh] OR "Relaxation Therapy"[Mesh] OR intervention*[tiab] OR program[tiab] OR programs[tiab] OR programme*[tiab] OR training[tiab] OR trial*[tiab] OR workshop*[tiab] OR course*[tiab] OR module*[tiab] OR curricul*[tiab] OR mindfulness[tiab] OR meditat*[tiab] OR relaxation[tiab] OR biofeedback[tiab] OR yoga[tiab] OR psychoeducat*[tiab] OR "cognitive behavio*"[tiab] OR counsel*[tiab] OR mentor*[tiab] OR "peer support"[tiab] OR "animal assisted"[tiab] OR "animal-assisted"[tiab] OR "e-learning"[tiab] OR eLearning[tiab] OR digital[tiab] OR app[tiab] OR apps[tiab] OR online[tiab] |
| #4 Combined | #1 AND #2 AND #3 |
| #5 Date | #4 AND ("2015/01/01"[Date - Publication]: "2026/06/30"[Date - Publication]) |

**S1.3 Embase (Embase.com)**

Platform: Embase.com (Elsevier); searched 30 June 2026; yield: 1,391; limits: publication years 2015–2026; conference abstracts excluded; no language restriction.

| **Line** | **Search string** |
| --- | --- |
| #1 Population | 'dental student'/exp OR 'dental student*':ti,ab OR 'dentistry student*':ti,ab OR (('dental':ti,ab OR 'dentistry':ti,ab OR 'oral health':ti,ab) AND ('student*':ti,ab OR 'undergraduate*':ti,ab)) |
| #2 Outcomes | 'mental stress'/exp OR 'burnout'/exp OR 'psychological resilience'/exp OR 'wellbeing'/exp OR stress*:ti,ab OR distress*:ti,ab OR burnout:ti,ab OR resilien*:ti,ab OR wellbeing:ti,ab OR 'well being':ti,ab OR anxiety:ti,ab OR coping:ti,ab |
| #3 Intervention | 'mindfulness'/exp OR 'meditation'/exp OR 'relaxation training'/exp OR intervention*:ti,ab OR program*:ti,ab OR training:ti,ab OR trial*:ti,ab OR workshop*:ti,ab OR module*:ti,ab OR curricul*:ti,ab OR mindfulness:ti,ab OR meditat*:ti,ab OR relaxation:ti,ab OR biofeedback:ti,ab OR 'peer support':ti,ab OR mentor*:ti,ab OR 'e-learning':ti,ab OR digital:ti,ab |
| #4 Combined | #1 AND #2 AND #3 AND [2015-2026]/py NOT 'conference abstract'/it |

**S1.4 Remaining sources (adapted syntax)**

| **Line** | **Platform** | **Search string** |
| --- | --- | --- |
| Scopus | Scopus.com (Elsevier); searched 30 June 2026; yield: 987; limits: publication year after 2014; no language restriction. | TITLE-ABS-KEY(("dental student*" OR "dentistry student*" OR "dental hygiene student*" OR "oral health student*") AND (stress* OR distress* OR burnout OR resilien* OR wellbeing OR "well-being" OR anxiety OR coping) AND (intervention* OR program* OR training OR trial* OR workshop* OR curricul* OR mindfulness OR meditat* OR relaxation OR biofeedback OR "peer support" OR mentor* OR "e-learning" OR digital)) AND PUBYEAR > 2014 |
| Web of Science | Clarivate Web of Science; searched 30 June 2026; yield: 764; timespan: 1 January 2015–30 June 2026; no language restriction. | TS=(("dental student*" OR "dentistry student*" OR "dental hygiene student*" OR "oral health student*") AND (stress* OR distress* OR burnout OR resilien* OR wellbeing OR "well-being" OR anxiety OR coping) AND (intervention* OR program* OR training OR trial* OR workshop* OR curricul* OR mindfulness OR meditat* OR relaxation OR biofeedback OR "peer support" OR mentor* OR "e-learning" OR digital)); Timespan: 2015-01-01 to 2026-06-30 |
| CENTRAL | Cochrane Library (Wiley); searched 30 June 2026; yield: 213; limits: Trials; publication years 2015–2026; no language restriction. | ([mh "Students, Dental"] OR "dental student*":ti,ab,kw) AND (stress* OR burnout OR resilien* OR wellbeing OR anxiety OR coping):ti,ab,kw; limits: publication year 2015–2026, Trials |
| ERIC (EBSCO) | EBSCOhost; searched 30 June 2026; yield: 121; date limiter: 1 January 2015–30 June 2026; no language restriction. | (DE "Dental Students" OR TI "dental student*" OR AB "dental student*") AND (TI (stress* OR burnout OR resilien* OR wellbeing OR coping) OR AB (stress* OR burnout OR resilien* OR wellbeing OR coping)) AND (TI (intervention* OR program* OR training OR curricul*) OR AB (intervention* OR program* OR training OR curricul*)); limiter: 20150101–20260630 |
| PsycINFO (Ovid) | Ovid; searched 30 June 2026; yield: 182; limits: publication years 2015–2026; no language restriction. | ((dental student* or dentistry student* or dental hygiene student*).ti,ab. or exp Dental Education/) and (stress* or distress* or burnout or resilien* or wellbeing or well-being or anxiety or coping).ti,ab. and (intervention* or program* or training or trial* or mindfulness or meditat* or relaxation or biofeedback or mentor* or peer support or e-learning or digital).ti,ab.; limit to yr="2015-2026" |
| ClinicalTrials.gov | ClinicalTrials.gov; searched 30 June 2026; yield: 24. | Condition/disease: stress OR burnout OR resilience; Other terms: dental students; Study start: 01/01/2015–06/30/2026 |
| WHO ICTRP | WHO International Clinical Trials Registry Platform; searched 30 June 2026; yield: 13. | dental students AND (stress OR burnout OR resilience) |

# Appendix S2. Risk-of-bias assessments

Randomized parallel-group trials and the study reporting random group allocation were assessed with RoB 2. Non-randomized controlled studies were assessed with ROBINS-I. Crossover-type or repeated-exposure studies without a randomized sequence were retained within ROBINS-I because confounding and selection were central threats; intervention order, period effects, carryover or washout, time-varying stressors, and paired/repeated-measures analysis were additionally considered. Uncontrolled studies were assessed using criteria adapted from the NHLBI/NIH Quality Assessment Tool for Before-After Studies With No Control Group. No numeric quality score was calculated. Judgments concern the principal self-reported stress, burnout, or resilience outcome; abbreviations are defined beneath each table.

**Table S2.1.** RoB 2 judgments for studies with random allocation.

| **Study** | **D1** | **D2** | **D3** | **D4** | **D5** | **Overall** |
| --- | --- | --- | --- | --- | --- | --- |
| Hayati et al. (2026)[1] | Some concerns | Some concerns | Some concerns | Some concerns | Some concerns | High |
| Kanderis Lane et al. (2021)[2] | Some concerns | Some concerns | Low | Some concerns | Some concerns | Some concerns |
| Repo et al. (2022)[3] | Low | Some concerns | Some concerns | Some concerns | Low | Some concerns |
| Smith et al. (2019)[4] | Some concerns | Some concerns | High | Some concerns | Some concerns | High |

RoB 2: D1, randomization process; D2, deviations from intended interventions; D3, missing outcome data; D4, measurement of the outcome; D5, selection of the reported result.

**Table S2.2.** ROBINS-I judgments for non-randomized controlled or crossover studies.

| **Study** | **D1** | **D2** | **D3** | **D4** | **D5** | **D6** | **D7** | **Overall** |
| --- | --- | --- | --- | --- | --- | --- | --- | --- |
| Lin et al. (2026)[5] | Serious | Moderate | Low | Moderate | Moderate | Moderate | Moderate | Serious |
| Aboalshamat et al. (2020)[6] | Serious | Serious | Low | Moderate | Moderate | Moderate | Moderate | Serious |
| Alzahem et al. (2015)[7] | Serious | Moderate | Low | Moderate | Low | Moderate | Moderate | Serious |
| Felszeghy et al. (2024)[8] | Serious | Moderate | Moderate | Moderate | Low | Moderate | Moderate | Serious |

ROBINS-I: D1, confounding; D2, participant selection; D3, classification of interventions; D4, deviations from intended interventions; D5, missing data; D6, outcome measurement; D7, selection of the reported result.

**Table S2.3.** Credibility appraisal of uncontrolled studies.

| **Study** | **Principal credibility limitations** | **Overall** |
| --- | --- | --- |
| Anishchuk et al. (2026)[9] | No control; post-test denominator unclear; short horizon | Serious concerns |
| Patarathipakorn et al. (2025)[10] | Baseline stress selection; maturation/examination-cycle effects; internally inconsistent study dates | Serious concerns |
| Adams (2017)[11] | Self-selected service users; counseling dose confounded with clinical need; no uniform endpoint | Serious concerns |
| Fourie et al. (2026)[12] | 65% follow-up loss and very low app use; complete-case result highly unstable | Serious concerns |
| González and Quezada (2016)[13] | Five self-referred cases; no counterfactual; therapist and outcome measurement not blinded | Serious concerns |
| Islam et al. (2026)[14] | Same-day pre/post measurement; no control; expectancy and repeated-testing effects | Serious concerns |

**S2.4** Study-level support for overall judgments.

| **Study** | **Principal credibility limitations** |
| --- | --- |
| Hayati et al.[1] | Random allocation asserted within a quasi-experimental report, but sequence generation, concealment, missing-data handling, and prespecified reporting were insufficiently described. |
| Kanderis Lane et al.[2] | Random-number assignment was described; participants and assessors were not blinded, the sample was small, and no prespecified analysis plan was identified. |
| Repo et al.[3] | Registered trial with clear groups; contamination, unequal online-group size, some attrition, and self-reported outcomes raised concerns. Dental subgroup outcomes were not separable. |
| Smith et al.[4] | Allocation method and concealment were unclear, initial response was 47.6%, and follow-up loss was differential; the self-reported endpoint was unblinded. |
| Lin et al.[5] | Voluntary training and non-participant controls create serious confounding by motivation, baseline stress, help-seeking, and curricular stage. |
| Aboalshamat et al.[6] | Students selected whether to receive coaching; an all-female single-school sample and incomplete follow-up amplify selection and confounding. |
| Alzahem et al.[7] | The staggered waitlist compared different academic-year cohorts and did not use randomized sequence allocation. A conventional washout was neither reported nor conceptually adequate because stress-management skills could persist; cohort, period, and carryover effects therefore cannot be separated confidently, although repeated group-by-time analyses were reported. |
| Felszeghy et al.[8] | Coping strategies and their order were self-selected across repeated simulation sessions. No washout was reported, and learned breathing or music routines could persist into later non-use periods; paired/repeated self-ratings did not fully isolate order, period, or carryover effects. |

Outcome bodies containing randomized evidence started at high certainty; outcomes supported only by non-randomized or uncontrolled evidence started at low certainty. In accordance with the reported protocol deviation, one certainty judgment was made per outcome because the evidence was too sparse for separate intervention-category and design-specific bodies. Controlled evidence anchored causal interpretation, whereas uncontrolled findings were treated as supportive or hypothesis-generating. Domains were rated as not serious, serious (-1), or very serious (-2), with no rating below very low. No upgrading criterion was met.

**S2.5**. Outcome-level GRADE evidence profile and downgrading decisions.

| **Outcome** | **Starting certainty** | **Domain judgments and downgrades** | **Publication bias / other** | **Final certainty** |
| --- | --- | --- | --- | --- |
| Stress/  distress | High (randomized evidence available) | Risk of bias: very serious (-2), with no low-risk study and several influential high/serious-risk studies. Inconsistency: serious (-1), because controlled and within-group findings differed in direction and magnitude. Indirectness: serious (-1), because 102/619 participants came from an inseparable mixed cohort. Imprecision: serious (-1), because analyses were small and confidence intervals were often absent. | Not quantitatively assessable* | Very low |
| Burnout | Low (one uncontrolled study) | Risk of bias: serious (-1), because measurement was same-day, uncontrolled, and unblinded. Inconsistency: not serious for the single study. Indirectness: not serious. Imprecision: serious (-1), because n=69, with no comparator, confidence interval, or follow-up. | Not quantitatively assessable* | Very low |
| Resilience | Low (non-randomized evidence only) | Risk of bias: serious (-1), owing to self-selection/confounding and incomplete denominators. Inconsistency: serious (-1), because findings ranged from a medium controlled effect to little/ or no effect. Indirectness: not serious. Imprecision: serious (-1), because analyses were small and confidence intervals largely unavailable. | Not quantitatively assessable* | Very low |

** Funnel plots and regression tests were not feasible because no meta-analysis contained at least 10 comparable studies, effect estimates and variances were frequently unavailable, and designs, measures, and time points differed substantially. Trial registries were searched, but residual publication and selective-outcome-reporting bias cannot be excluded. No separate publication-bias downgrade or upgrading criterion was applied.*

# Appendix S3. Detailed characteristics and findings of all included studies

Study sample sizes and outcome denominators are reported separately. NR, not reported; ACT, Acceptance and Commitment Therapy; BDS, Bachelor of Dental Surgery; CBT, cognitive-behavioral therapy. The 14 study-reported sample sizes sum to 750, but this value is not a pooled analysis denominator or a dental-student-only total: it includes all 102 participants in the mixed health-professions Repo et al. trial, in which dentistry-specific numbers and outcomes were not separable; 648 participants came from dental/oral-health-only cohorts. The stress/psychological-distress evidence comprises 12 studies with reported sample sizes totaling 619; the burnout evidence comprises one study with 69 participants; and the resilience evidence comprises three studies totaling 206. Participants may contribute to more than one outcome category, and outcome-specific analyses were frequently smaller because of attrition or missing data.

**Table S3.1** Design, participants, intervention, comparator, and follow-up.

| **Study; country** | **Design** | **Participants** | **Intervention and dose** | **Comparator** | **Follow-up; retention/funding** |
| --- | --- | --- | --- | --- | --- |
| Anishchuk et al. (2026)[9]; Ireland | Uncontrolled curriculum-embedded pre–post evaluation | 62 baseline respondents; post-test denominator not clearly reported; Later-year dental undergraduates; 80% female at baseline | ResiliDents asynchronous eLearning module on resilience and coping; embedded in curriculum | None | Immediate post-module; Outcome-specific denominator unclear; SATLE public funding |
| Lin et al. (2026)[5]; Germany | Non-randomized controlled pre–post study | 56 (27 training; 29 non-participant controls); Preclinical and clinical dental students | Two-day online resilience training tailored to dental-school stressors | Non-participant student controls | Six-month study window; Not clearly reported in the accessible report; funding not stated |
| Patarathipakorn et al. (2025)[10]; Thailand | Uncontrolled quasi-experimental study | 31 selected; 28 completed; First-year dental students with T-PSS-10 scores 1–26; 67.9% female among completers | Anapanasati meditation: four workshops plus 60 min/day walking and sitting meditation for 14 weeks | None | Four weeks after intervention; 3/31 (9.7%) did not complete; funding not clearly reported |
| Aboalshamat et al. (2020)[6]; Saudi Arabia | Non-randomized controlled interventional study | 88 analyzed (44 intervention; 44 control); 97 began; Female BDS students, years 2–6 | One introductory lecture plus five weekly 15-min telephone life-coaching sessions using the GROW model | No coaching | Immediately after five weeks; 9/97 (9.3%) did not complete; no specific funding reported |
| Adams (2017)[11]; United States | Uncontrolled embedded-service evaluation | 55 students; 251 appointments; Predoctoral dental students using an embedded counselor | Individual counseling embedded within the dental school; mean 4.5 appointments/student | None | Repeated contacts over three semesters; no uniform endpoint; Service-use denominators varied by measure; funding not reported |
| Alzahem et al. (2015) [7]; Saudi Arabia | Non-randomized waitlist crossover | 31 (15 third-year; 16 second-year); all male; Second- and third-year dental students | Dental Education Stress Management program: three 90-min sessions over three weeks (psychoeducation, breathing, CBT, time management) | Staggered waitlist cohort | Two weeks after each cohort completed the program; No attrition reported; funding not clearly reported |
| Felszeghy et al. (2024)[8]; Finland | Within-participant crossover evaluation | 31 (24 female; 7 male); Volunteer third-year dental students | Self-selected background music and deep belly breathing during fixed-prosthodontic simulation exercises | Practice sessions without the coping strategies | End of preclinical course / immediate exercise ratings; All 31 represented in main table; funding not clearly reported |
| Fourie et al. (2026)[12]; South Africa | Uncontrolled prospective pilot | 31 enrolled; 26 baseline; 9 follow-up; Oral-hygiene and second- to fourth-year dentistry students | Medito smartphone app, 10 min/day for eight weeks | None | Eight weeks; 9/26 baseline participants completed (65% loss); no funding |
| González and Quezada (2016)[13]; Chile | Uncontrolled A-B-C multiple-case series | 5; Dental students who voluntarily sought psychological care | Eight-session individual cognitive-behavioral intervention | None | One month after the last session; All five cases described; CONICYT support reported |
| Hayati et al. (2026)[1]; Indonesia | Three-arm quasi-experimental study reporting random group allocation | 120; Dental students | 40-Hz isochronic tones for 30 min/day or 10 min/day for 14 days | No-tone control | Immediate post-intervention; Not clearly reported in the abstract; funding not stated |
| Islam et al. (2026)[14]; Pakistan | Uncontrolled mixed-methods quasi-experimental study | 69 (37 female; 32 male); Final-year BDS students | Two 2-h mindfulness/life-skill-building sessions delivered on one day | None | Immediate post-session; All 69 completed; no specific funding |
| Kanderis Lane et al. (2021)[2]; United States | Non-blinded randomized controlled trial | 32 (16 yoga; 16 control); all female; First-year entry-level dental-hygiene students | Six 15-min video-guided yoga/breathing sessions immediately before final examinations | Usual pre-examination routine | End of finals week; One control baseline PSS missing; post-trial data for all 32; no funding stated |
| Repo et al. (2022)[3]; Finland | Three-arm randomized controlled trial | 102 (40 face-to-face; 22 web ACT; 40 control); Mixed medicine, dentistry, psychology, and logopaedics cohort; dental results not separable | Mindfulness Skills for Students course or web-based Student Compass ACT program | Student mental-health support as usual | Post-intervention and four months; Immediate analyses: 39/40, 20/22, 38/40; registered NCT03669016; faculty funded |
| Smith et al. (2019)[4]; New Zealand | Randomized active-control trial | 40 baseline respondents (20/group) from 84 invited; Fourth-year BDS students | 4.5-min video teaching cognitive reappraisal | 4.5-min generic stress-management video | Reported as three weeks; methods also state surveys were eight weeks apart; 47.6% baseline response; differential follow-up loss; internal faculty funding |

**Table S3.2** Outcome measures and principal numerical findings.

| **Study; country** | **Outcome measures** | **Principal numerical finding** | **Retention/funding note** |
| --- | --- | --- | --- |
| Anishchuk et al. (2026)[9] | CD-RISC-25 before and after the module | CD-RISC-25 63.84 (SD 15.69) to 68.87 (SD 18.06), +7.9%, not statistically significant; coping subscale improved (p<0.01). | Outcome-specific denominator unclear; SATLE public funding |
| Lin et al. (2026)[5] | Resilience, perceived stress, life satisfaction, health behavior; online surveys | Medium group-time effect on resilience (F(1,54)=4.93, p=0.031, η²=0.084); lower subjective stress and higher life satisfaction were reported; health-behavior findings were inconclusive. | Not clearly reported in the accessible report; funding not stated |
| Patarathipakorn et al. (2025)[10] | T-PSS-10 at baseline, week 14, and week 18; group discussion | T-PSS-10 17.7 to 9.8 to 8.4 (p<0.001). Large within-person changes cannot be separated from time, selection, or examination-cycle effects. | 3/31 (9.7%) did not complete; funding not clearly reported |
| Aboalshamat et al. (2020)[6] | DASS-21, RS-14, perceived wellness, goal approach; pre/post | Between-group time effects favored coaching for depression (p=0.026, ηp²=0.026) and stress (p=0.010, ηp²=0.074); anxiety and resilience did not improve versus control. | 9/97 (9.3%) did not complete; no specific funding reported |
| Adams (2017)[11] | CCAPS-34 and Outcome Rating Scale across counseling contacts | At intake, 45/55 (82%) had a clinically significant score on at least one CCAPS-34 subscale; more appointments were associated with better overall functioning, without a causal comparator. | Service-use denominators varied by measure; funding not reported |
| Alzahem et al. (2015)[7] | DES and PSM-9 at three time points | Group-time interactions favored the program for DES (F(2,58)=10.36, ηp²=0.22) and PSM-9 (F(2,58)=8.19, ηp²=0.04); effects persisted for at least two weeks. | No attrition reported; funding not clearly reported |
| Felszeghy et al. (2024)[8] | GHQ-12, PHQ-4, and VAS-Anxiety | GHQ-12 stress decreased in women (p=0.025) but not men; music and breathing reduced VAS fear/stress, although a clear contrast with non-use was not demonstrated. | All 31 represented in main table; funding not clearly reported |
| Fourie et al. (2026)[12] | PSS-10, 0–10 self-rated stress, app use | PSS-10 25.81 (SD 7.96) to 20.33 (SD 8.31), p=0.50; self-rated stress 7.54 to 5.78, p=0.50. Mean use was only 53 min over about six days. | 9/26 baseline participants completed (65% loss); no funding |
| González and Quezada (2016)[13] | OQ-45.2, Dental Environment Stress questionnaire, exit interview | All five reported lower dental-environment stress; two participants moved from dysfunctional to normal OQ-45.2 scores; the remaining three stayed in the normal range. | All five cases described; CONICYT support reported |
| Hayati et al. (2026)[1] | DASS before and after 14 days | The 30-min group improved within group for anxiety, depression, and stress (all p<0.05); post-intervention group differences were reported for anxiety and depression, not stress. | Not clearly reported in the abstract; funding not stated |
| Islam et al. (2026)[14] | CBI-SS before and immediately after; stakeholder focus groups | Personal burnout (mean difference 1.62, p=0.003) and study-related burnout (1.88, p=0.005) decreased; colleague- and teacher-related domains did not change significantly. | All 69 completed; no specific funding |
| Kanderis Lane et al. (2021)[2] | PSS-10, blood pressure, pulse | PSS fell within the yoga group by 1.8 points (p<0.001), but post-trial PSS did not differ from control (p=0.68); several acute physiological measures improved. | One control baseline PSS missing; post-trial data for all 32; no funding stated |
| Repo et al. (2022)[3] | CORE-OM primary; stress, well-being, mindfulness, hair cortisol secondary | Primary CORE-OM differences were not significant (face-to-face p=0.076; web p=0.516; pooled p=0.067); functioning/social interaction favored face-to-face and pooled groups (p=0.028). No primary group difference remained at four months. | Immediate analyses: 39/40, 20/22, 38/40; registered NCT03669016; faculty funded |
| Smith et al. (2019)[4] | Modified 20-item Dental Environment Stress questionnaire | Mean DES change favored reappraisal (+3.10 vs −1.06), but the between-group difference was not statistically significant (p=0.076). | 47.6% baseline response; differential follow-up loss; internal faculty funding |

The 14 study-reported sample sizes sum to 750, but this value is neither a pooled analysis denominator nor a dental-student-only total. It includes all 102 participants in the mixed health-professions Repo et al. trial, in which the number and outcomes of dental students were not separable; 648 participants came from dental/oral-health-only cohorts, and an unknown subset of the 102 mixed-cohort participants were dental students. The perceived-stress/ psychological-distress evidence comprises 12 studies with study-reported sample sizes totaling 619, including the same 102 mixed-cohort participants; the burnout evidence comprises one study with 69 participants; and the resilience evidence comprises three studies totaling 206. Participants may contribute to more than one outcome category, and outcome-specific analyses were frequently smaller because of attrition or missing data.

# Reference

1.	Hayati AK, Sulisetyawati TIB, Haryanto J, Aljunaid MA, Al-Ghazaly F, Sari MR: **The Effectiveness of Isochronic Tones Against Anxiety, Depression, and Stress in Dental Students**. *North American Journal of Psychology* 2026, **26**(2):826.

2.	Kanderis Lane CL, Gurenlian JR, Freudenthal J, Denner PR: **A 15-Minute Yoga Intervention to Reduce Entry-Level Dental Hygiene Student Stress**. *J Dent Hyg* 2021, **95**(2):63–70.

3.	Repo S, Elovainio M, Pyörälä E, Iriarte-Lüttjohann M, Tuominen T, Härkönen T, Gluschkoff K, Paunio T: **Comparison of two different mindfulness interventions among health care students in Finland: a randomised controlled trial: S. Repo et al**. *Advances in Health Sciences Education* 2022, **27**(3):709–734.

4.	Smith A, Scott I, Ratnayake J, Newsham-West K, Cathro P: **An Intervention Study: Does a Cognitive Reappraisal Technique Reduce the Perceived Stress in Fourth-Year Dental Students in New Zealand?** *Int J Dent* 2019, **2019**(1):5864591.

5.	Lin LX, Keul C, Fischer MR, Kiesewetter J: **Stress and Resilience Development in German Dental Students**. *Eur J Dent Educ* 2026.

6.	Aboalshamat K, Al-Zaidi D, Jawa D, Al-Harbi H, Alharbi R, Al-Otaibi S: **The effect of life coaching on psychological distress among dental students: interventional study**. *BMC Psychol* 2020, **8**(1):106.

7.	Alzahem AM, Van der Molen HT, De Boer BJ: **Effectiveness of a dental students stress management program**. *Health Professions Education* 2015, **1**(1):34–42.

8.	Felszeghy S, Kurki P, Liukkonen M, Suominen AL, Huhtela O: **The combination of listening to background music and deep breathing as a stress reduction strategy during dental preclinical study**. *J Dent Educ* 2024, **88**(3):342–347.

9.	Anishchuk S, Phadraig CM, Roche C, Kubacki A, Sullivan D, Howell Y: **ResiliDents: Development and Evaluation of Resilience Module in Dental Undergraduate Curriculum**. *Eur J Dent Educ* 2026, **30**(1):87–97.

10.	Patarathipakorn O, Pawa KK, Sritipsukho P, Tansuhaj K, Bhamarapravatana K, Suwannarurk K: **Aanapanasati Meditation and Stress Reduction Among Health Science University Students**. *The Open Public Health Journal* 2025, **18**(1).

11.	Adams DF: **The Embedded Counseling Model: An Application to Dental Students**. *J Dent Educ* 2017, **81**(1):29–35.

12.	Fourie J, Kotze C, Hendricks H, Bhayat A: **Feasibility of a Digital Mindfulness Intervention for Stress Reduction Among Dental Students: A Pilot Study**. *South African Dental Journal* 2026, **81**(1):13–17.

13.	Gònzalez G, Quezada VE: **A brief cognitive-behavioral intervention for stress, anxiety and depressive symptoms in dental students**. *Research in Psychotherapy: Psychopathology, Process and Outcome* 2016, **19**(1).

14.	Islam A, Rehman J, Mujeeb S, Javed R, Hashmi U, Ali TS: **Investigating the effectiveness of structured mindfulness sessions in mitigating burnout among final-year dental students: A mixed-methods analysis**. *PloS one* 2026, **21**(2):e0342017.
